# Anomalous Branch of the Left Hepatic Artery With Pericardial, Diaphragmatic, Splenic and Gastric Supply During Selective Internal Radiotherapy (SIRT)

**DOI:** 10.7759/cureus.20373

**Published:** 2021-12-12

**Authors:** Naushad H Karim, Jon Bell, Damian Mullan, Jeremy Lawrance, Pavan Najran

**Affiliations:** 1 Interventional Radiology, The Christie National Health Service Foundation Trust, Manchester, GBR; 2 Radiology and Interventional Radiology, The Christie National Health Service Foundation Trust, Manchester, GBR; 3 Radiology, The Christie National Health Service Foundation Trust, Manchester, GBR

**Keywords:** single photon emission tomography (spect), diaphragm, stomach, spleen, pericardium, anomalous left hepatic artery branch, selective internal radiotherapy (sirt)

## Abstract

Selective internal radiotherapy (SIRT) is an established modality for the treatment of hepatic malignancy. The procedure is normally carried out in two parts. The first part involves a planning or "work-up" angiogram to delineate anatomy and plan safe yttrium-90 (Y90) delivery, and the second part for the administration of the Y90 microspheres. The work-up angiogram has three main purposes including delineation of hepatic and tumor vascular anatomy, which might influence the administration of the microbeads, identification, and embolization of blood vessels, which may complicate treatment or contribute to non-target Y90 microsphere deposition and administration of technetium 99 (metastable) labeled macroaggregated albumin (^99m^TcMAA) at the planned administration points prior to the same day single-photon emission computed tomography (SPECT) or planar SPECT to identify sites of ^99m^TcMAA uptake. We present the case of a SIRT procedure that demonstrated an anomalous artery arising from the left hepatic artery with supply to the pericardium, diaphragm, fundus of the stomach, and spleen. This is a rare vascular variant that highlights the importance of thorough assessment of both the planning angiograms and SPECT CT for the presence of anatomical variants and abnormal extrahepatic ^99m^TcMAA uptake to help reduce the need to recall patients for repeat work-up procedures.

## Introduction

Selective internal radiotherapy (SIRT) is an established modality for the treatment of hepatic malignancy, either primary liver cancer or liver metastases. The therapy involves the administration of microbeads containing yttrium-90 (Y90), which is a beta emitter. The microbeads are normally either made of resin (Sirspheres; Sirtex Medical Limited, Sydney, Australia) or glass (Theraspheres; Boston Scientific, Marlborough, MA, USA).

The procedure is normally carried out as a two-part procedure with the first part involving a planning or "work-up" angiogram to delineate anatomy and plan safe Y90 delivery, and the second part for the administration of the Y90 microspheres [1]. In most instances, these are not done on the same day, however, single-day SIRT planning and administration procedures have been described [2].

The planning procedure/work-up has three main purposes [1]. The first is to delineate and define hepatic and tumor vascular anatomy, which might influence the administration of the microbeads. The variance of arterial supply to the liver is common with portions of the liver not uncommonly supplied from the superior mesenteric artery (SMA) or left gastric artery [3]. Formal angiography of the SMA and celiac axis is performed during the SIRT work-up to look for anatomical variance. This is followed by selective angiograms of all vessels identified as supplying the liver, which can include the SMA, left gastric, common hepatic, hepatic artery proper, and right and left hepatic arteries.

The second purpose of the work-up is to identify and decide whether there is a need to embolize vessels, which might complicate treatment or contribute to non-target Y90 microsphere deposition during the subsequent treatment [4]. In recent years, there has been a shift toward avoiding vessel embolization unless absolutely necessary [5]. Common branches of concern, which are at risk of contributing to non-target embolization include the right gastric artery and gastroduodenal artery. When the administration of microspheres to the left hepatic artery is required, it is important to keep in mind that the left hepatic artery more commonly gives rise to anatomical variants [3]. These can include branches of the gastrohepatic trunk and accessory gastric, esophageal and phrenic arteries. These could preclude safe administration of the microspheres and must be identified and, if necessary embolized prior to Y90 administration. If there are any concerns of extrahepatic contribution to tumor vasculatures such as from the right inferior phrenic artery or intercostal arteries, then these too are embolized. Additional embolization of intrahepatic arteries may also be carried out to optimize tumoral blood flow and reduce procedural complexity [4]. At the end of this, the operator should have a firm understanding of the vascular anatomy, flow dynamics and likely points of administration of Y90.

The third purpose of the work-up is to deliver technetium 99 (metastable) labeled macroaggregated albumin (^99m^TcMAA) at the planned administration points prior to the same day single-photon emission computed tomography (SPECT) or planar SPECT to identify sites of ^99m^TcMAA uptake. ^99m^TcMAA is used as a surrogate for Y90 to confirm that the injected ^99m^TcMAA is delivered only to the liver with no concerning extrahepatic uptake. It is also used to estimate lung shunt fraction as some lung uptake is to be expected. If the lung shunt fraction exceeds a threshold dose (commonly 20% of total dose), it may be unsafe to proceed with Y90 treatment due to the risk of radiation pneumonitis [6,7]. Some normal variant extrahepatic uptake may be seen, for example, within the stomach, thyroid, and salivary glands due to degradation of the ^99m^TcMAA and release of free technetium into the bloodstream [8].

When abnormal extrahepatic uptake is identified, a repeat work-up with the knowledge of the anatomical distribution of the extrahepatic ^99m^TcMAA can allow targeted interrogation of potential sources, and in some cases allow embolization of aberrant vessels in subsequent treatment.

In recent years, the role of the ^99m^TcMAA scan has evolved from a tool used to exclude extrahepatic uptake to a tool used to help plan individualized personalized dosimetry [7].

We present the case of a SIRT procedure that demonstrated an anomalous artery arising from the left hepatic artery with supply to the pericardium, diaphragm, fundus of the stomach, and spleen. This is a rare vascular variant, which is to our knowledge the first described case of cardiac uptake of ^99m^TcMAA in SIRT. It highlights the importance of thorough assessment of both the planning angiograms and SPECT CT for the presence of anatomical variants, and abnormal extrahepatic ^99m^TcMAA uptake, to help reduce the need to recall patients for repeat work-up procedures.

## Case presentation

**Figure 1:**
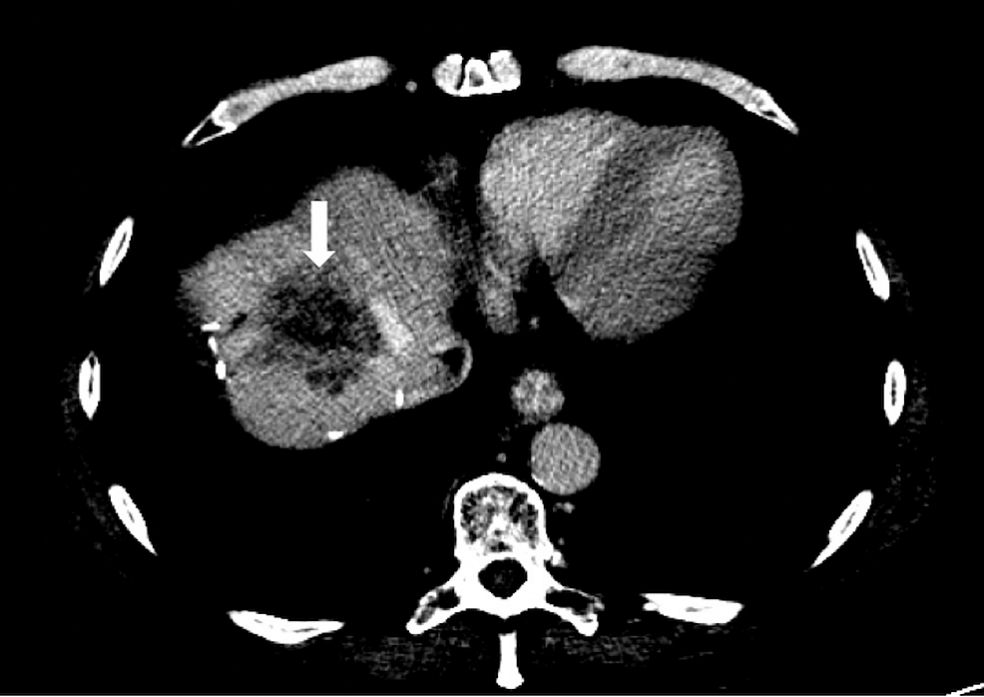
Axial CT abdomen in the portovenous phase in liver window demonstrating the segment 4a tumor (white arrow).

A 23-year-old male with colorectal cancer metastatic to the liver presented to our department for SIRT following discussion at the multi-disciplinary team (MDT). He had undergone previous right-sided liver resection and colonic resection with left iliac fossa stoma in situ. There was no background liver disease. The international normalizing ratio (INR) was 1.1, total bilirubin was 20 umol/L, albumin was 44 g/L, and creatinine was 68 umol/L. There was no presence of ascites. The Child-Pugh score was 5, and the model for end stage liver disease (MELD) score was 8. His recent CT abdomen demonstrated an inoperable segment 4a metastasis that was deemed unsuitable for percutaneous thermal ablation (Figure 1).

A planning procedure was carried out using a distal radial approach, with 5Fr ultimate 1 catheter (Merit Medical Systems Inc., South Jordan, UT, USA) and Progreat microcatheter (Terumo Medical, Tokyo, Japan). There was no collateral blood flow to the liver from the SMA. Celiac angiography demonstrated a native segment 4 and left lobe vessels. The right gastric artery was identified and coiled as it was felt to be close to the planned treatment injection point. A two-box treatment was adopted within the left lobe and segment 4 positions. ^99m^TcMAA was administered at these positions.

**Figure 2:**
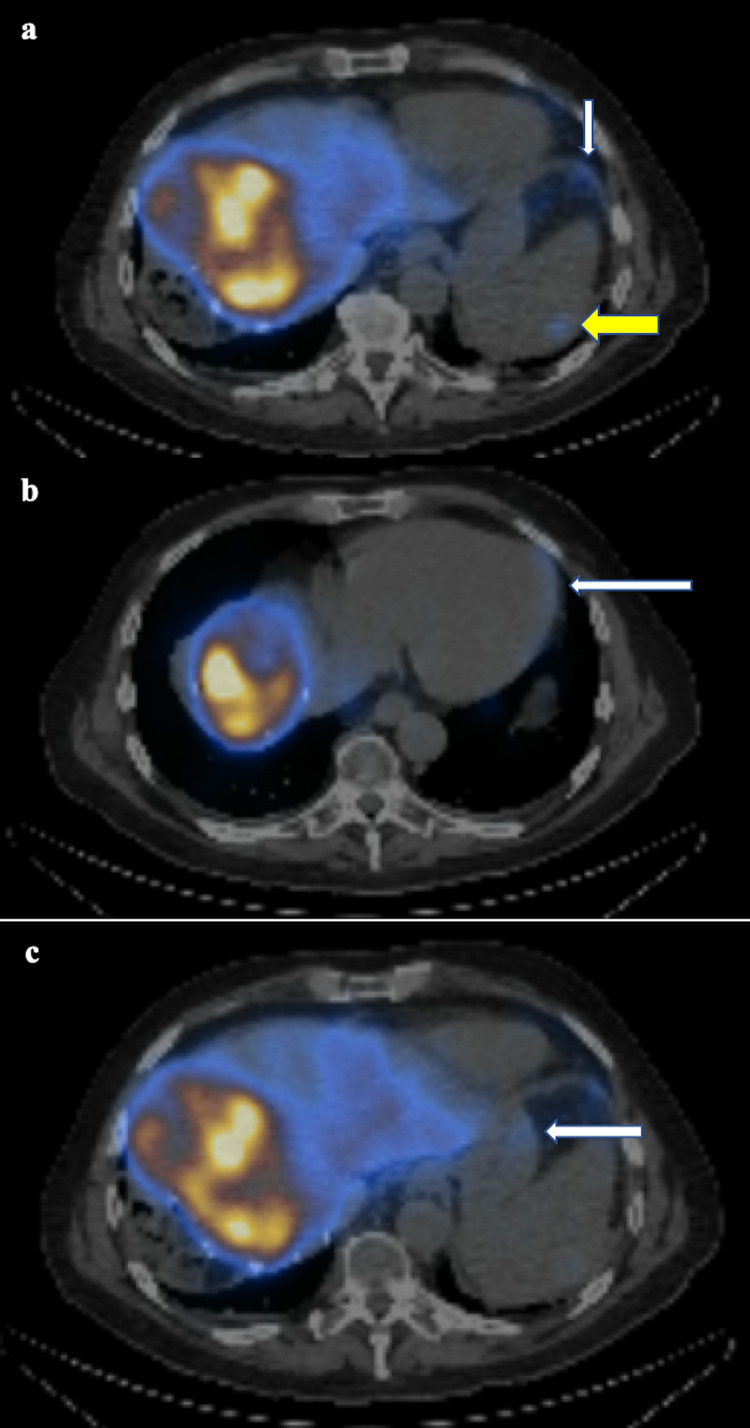
Axial SPECT images demonstrating small amounts of activity in the diaphragm (a, white arrow), spleen (a, yellow arrow), pericardium (b, white arrow), and stomach (c, white arrow). SPECT: single-photon emission computed tomography.

A SPECT CT was carried out after the administration of the ^99m^TcMAA. This demonstrated a small amount of activity in the diaphragm, stomach, spleen, and pericardium (Figure 2). This was calculated as 3.8% of the total activity. A post-procedural and retrospective review of the planning angiogram revealed a probable anatomical variant arising within the left lobe to account for the extrahepatic activity and it was felt that it could be coiled before treatment.

**Figure 3:**
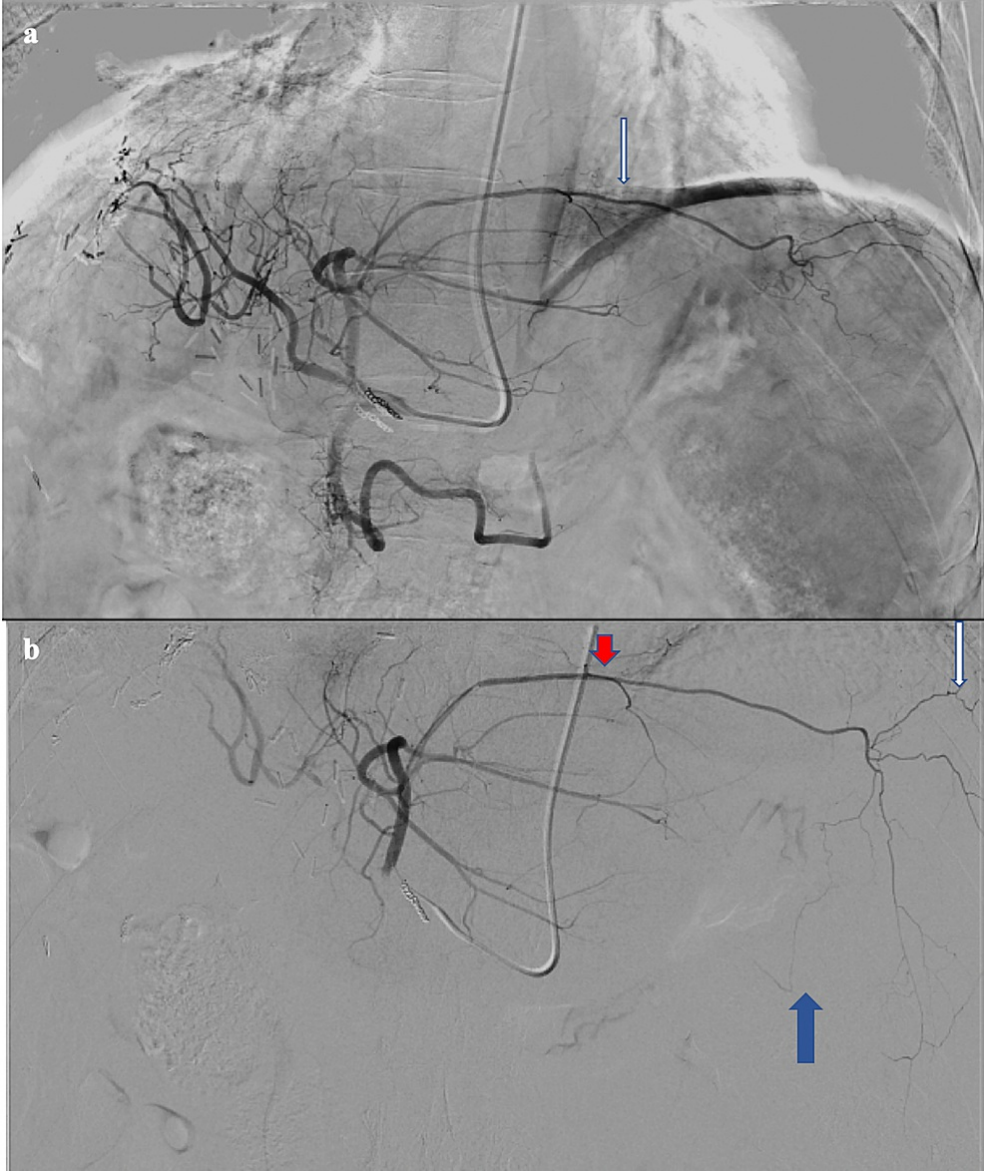
DSA angiogram from the left main hepatic artery demonstrating the anomalous branch (a, white arrow), and angiogram following selection of the anomalous branch with a microcatheter (b) demonstrating branches supplying the pericardium (red arrow), spleen (white arrow), and stomach (blue arrow). DSA: digital subtraction angiography.

A SIRT administration procedure was then carried out two weeks later. During the pre-administration angiogram of the left hepatic artery, the small blood vessel was again identified supplying the pericardium, diaphragm, stomach, and spleen (Figure 3).

**Figure 4:**
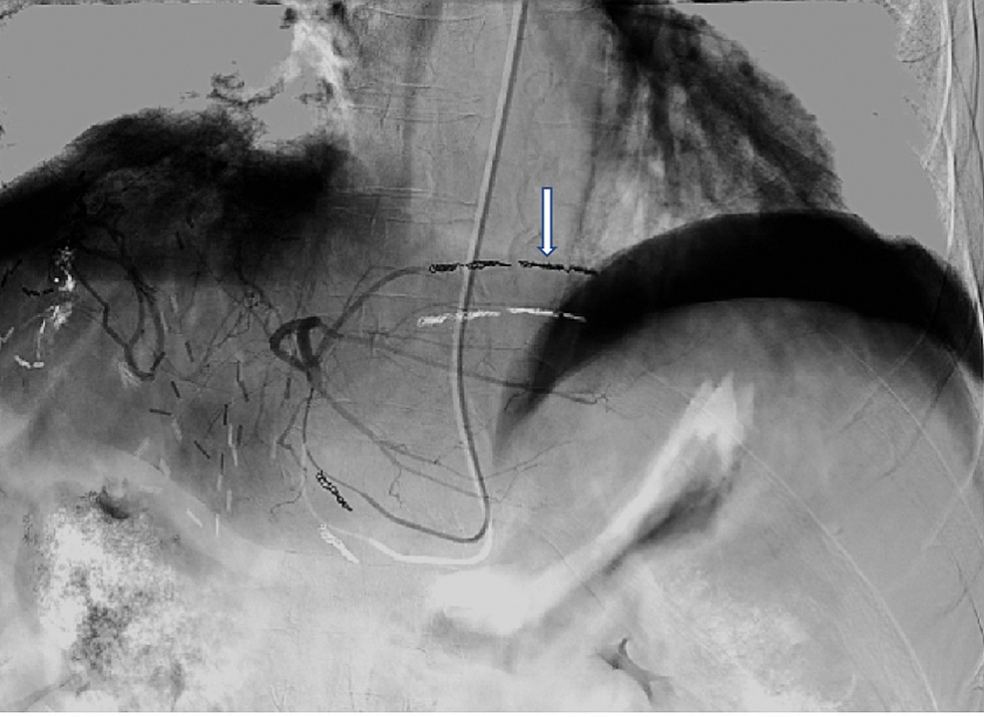
DSA angiogram after coiling demonstrating coils in the vessel (white arrow) with no contrast beyond in keeping with complete occlusion. DSA: digital subtraction angiography.

This was coiled off proximally, and a repeat angiogram demonstrated no flow within it (Figure 4).

**Figure 5:**
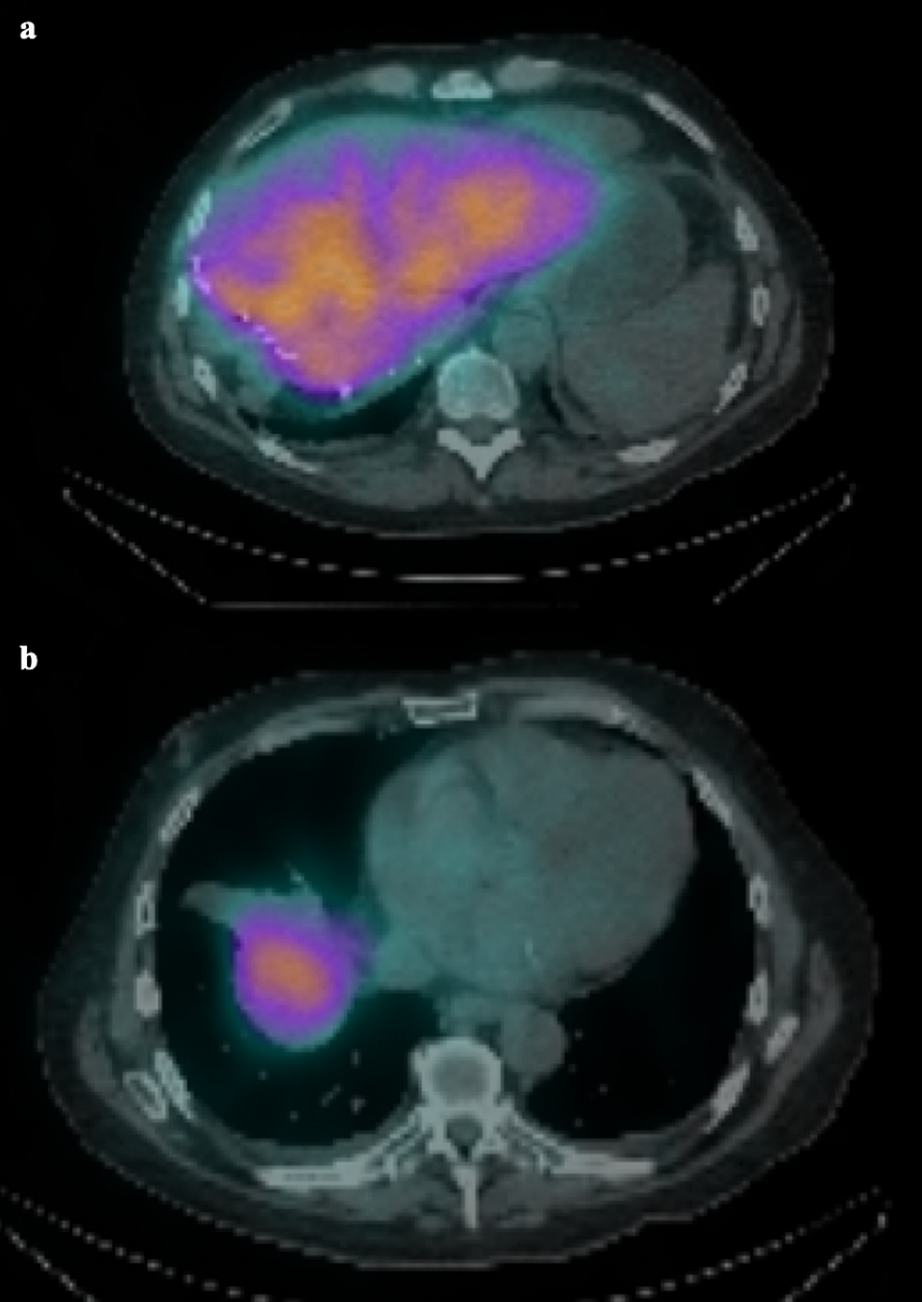
Bremstralung scan after the treatment at the level of the upper abdomen (a) and lower chest (b) demonstrating no activity in the previously seen areas.

The SIRT therapy was then administered at the planned points as before. SPECT CT after the procedure demonstrated satisfactory distribution within the liver parenchyma, with no extrahepatic distribution (Figure 5).

## Discussion

Extrahepatic areas of uptake on SPECT CT can be seen in or around the gastric wall and can often be attributed to ^99m^TcMAA degradation [9]. Important points on SPECT CT that could be indicative of variant vascular anatomy include areas of activity not attributable to free technetium from the degradation of MAA, such as the pericardium, diaphragm, and spleen. Minimizing the time between reconstitution of the ^99m^TcMAA kit in the nuclear medicine lab and administration in the interventional radiology (IR) suite, and in between administration and SPECT CT is also important in minimizing the degradation of ^99m^TcMAA that would result in free technetium into the bloodstream [6].

It is also important to image the entire extent of the liver during the planning angiograms to identify any accessory vessels arising from the hepatic arteries. This can be important for the left lobe, which can extend laterally up to the spleen, and is the most common source of extrahepatic variance supply [3].

To our knowledge, this is the first case to describe an anomalous artery arising from the left hepatic artery with supply to the pericardium.

In this case, although the extrahepatic dose fraction approximated by the SPECT CT was only 3.8%, had the microspheres been administered without identifying and coiling this anomalous artery, the patient may have developed potential complications including radiation pericarditis and gastritis. In this case, a retrospective review of the work-up angiogram allowed identification of the suspected variant and avoided the need for a repeat work-up.

## Conclusions

In recent years, the purpose of the ^99m^TcMAA has evolved from a scan to exclude extrahepatic uptake to a scan allowing personalized dosimetry calculations. This case highlights the importance of correlating ^99m^TcMAA SPECT CT findings with angiographic findings in identifying accessory vessels that could potentially lead to non-targeted deposition of the Y90 microspheres.
